# Monolithic integrated MXene supercapacitors may power future electronics

**DOI:** 10.1093/nsr/nwad020

**Published:** 2023-01-17

**Authors:** Yury Gogotsi

**Affiliations:** A. J. Drexel Nanomaterials Institute, and Department of Materials Science and Engineering, Drexel University, USA

Electrolytic capacitors and batteries are the largest components in portable electronics. Can they be replaced by fully integrated devices capable of performing functions ranging from energy storage to AC line-filtering and providing power pulses for communication? Wearable, flexible, transparent and epidermal electronics, micro-robots, and Internet of Things devices, will all require compact energy storage solutions. Monolithic, on-chip integrated MXene micro-supercapacitors (MSCs) free of separators and electric wires may offer a solution. More than a decade ago, carbon-based on-chip MSCs demonstrated their ability to operate at very high rates and harvest/deliver energy in milliseconds [1–3]. However, several barriers stood in their path. They had a fairly low energy density due to the double-layer charge storage mechanism, their internal resistance limited the number of devices that could be connected in series, and depositing electrolytes precisely on densely-packed MSCs while ensuring electrochemical isolation was a major challenge. In addition, electrochemical performance was often affected by multi-step microfabrication procedures [4].

**Figure 1.**
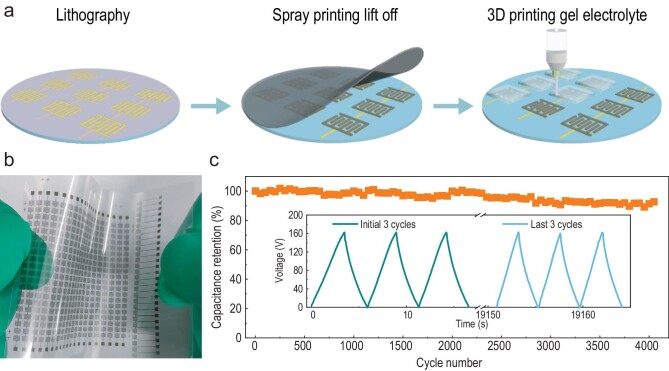
The fabrication and electrochemical characterization of monolithic integrated micro-supercapacitors. (a) A schematic of the fabrication process. (b) An array of MSCs on a flexible polyethylene terephthalate substrate and (c) cycling stability over 4000 cycles. Insets shows galvanostatic charge-discharge profiles. Reprinted with permission from Ref. [5].

Writing in *National Science Review*, Wang and colleagues address these critical issues by an innovative design of MXene MSCs [5]. They developed a high-throughput strategy combining multi-step lithographic patterning, spray printing of 2D Ti_3_C_2_T*_x_* MXene microelectrodes, and 3D printing of gel electrolyte for scalable manufacturing of interdigitated MSCs, simultaneously achieving superior cell number density and high performance of the entire array (Fig. 1). The use of MXene makes a big difference in device fabrication due to at least an order of magnitude higher electronic conductivity compared to carbon nanomaterials, redox ability of oxygen or OH-terminated atomic surface layers of Ti, and, last but not least, its solution processability [6,7] that leads to alignment of MXene flakes in-plane with the ions having the shortest possible path when traveling between the interdigitated electrodes. This leads to a low equivalent resistance of 0.3 Ohm/cm^2^, enabling operation at up to 500 V/s scan rate.

The monolithic integration of electrochemically isolated micro-supercapacitors in close proximity was achieved by leveraging high-resolution micropatterning techniques for microelectrode deposition and 3D printing for precise electrolyte deposition [5]. Super-dense microelectrode-arrays were fabricated, and each individual MXene-based MSC exhibited an extremely small footprint of 0.018 cm^2^, high areal and volumetric capacitance. A gel electrolyte ink enabled adjacent microcells to be electrochemically isolated at a close proximity of just 600 μm. Consequently, the authors obtained MSC arrays with a superior areal number density of 28 cells/cm^2^, a record areal output voltage of 75.6 V/cm^2^ and a volumetric energy density of 9.8 mWh/cm^3^, exceeding those of the previously reported integrated MSCs. An extremely high output voltage of 200 V was achieved by connecting 334 cells with an aqueous gel electrolyte. When 60 cells using an ionic liquid gel were connected in series, a voltage of 162 V was demonstrated. Those are record breaking values in terms of the number of cells and voltage, showing the versatility of the design.

There is still room for improvement, of course. The use of large-flake MXene as the current collector may eliminate gold and expand the voltage window in aqueaous electrolytes [7]. Increasing the electrode thickness would increase the areal energy and power density. However, even the currrent design reaches the energy density of thin-film batteries and approached the power density of electrolytic capacitors, thus offering many potential applications.

This innovative microfabrication strategy marks a great advance as a new technological platform for monolithic micropower sources that can be produced on a variety of substrates, made foldable and stretchable, and will aid in applications where compact integration and high systemic performance is demanded from energy storage units.
